# Retraction Note: Exploring MAP3K genes in gastric cancer: biomarkers, tumor microenvironment dynamics, and chemotherapy resistance

**DOI:** 10.1186/s41065-026-00747-x

**Published:** 2026-10-09

**Authors:** Senhui Wei, Ying Li, Jing Zhou, Yongming Xia

**Affiliations:** 1 Department of Gastroenterolog, Shenzhen Guangming District People’s Hospital, Shenzhen City, 518107 P.R. China; 2 Department of Hepatobiliary Gastrointestinal Surgery, Shenzhen Guangming District People’s Hospital, Shenzhen City, 518107 P. R. China

**Retraction Note: Hereditas 162, 15** **(2025)**

**https://doi.org/10.1186/s41065-025-00364-0**

The Editor-in-Chief has retracted this article. After publication, concerns were raised regarding the in vitro images presented in Fig. 10 if this article, where the patterns of cells and colonies do not appear consistent with natural cell growth and proliferation. Additionally, the uncut western blot gel presented in the Supplementary Material appears to show contradictory results, with two proteins probed in adjacent lanes with different results. It has also been noted that a number of references in this article do not appear to support the statements preceding the citations.

In addition, the BGC823, MGC803 and SGC7901 cell lines used in this study have been reported to be contaminated with HeLa cells, making them an unsuitable model for gastric cancer.

The Editor-in-Chief therefore no longer has confidence in the presented data.

The authors have not responded to correspondence regarding this retraction.

